# The tumor microenvironment in hepatocellular carcinoma: mechanistic insights and therapeutic potential of traditional Chinese medicine

**DOI:** 10.1186/s12943-025-02378-8

**Published:** 2025-06-10

**Authors:** Xiaojun Su, Xiuli Yan, Hui Zhang

**Affiliations:** 1 Institute of Interdisciplinary Integrative Medicine Research, Shanghai University of Traditional Chinese Medicine, Shanghai, 201203 China; 2 Yueyang Hospital of Integrated Traditional Chinese and Western Medicine, Shanghai University of Traditional Chinese Medicine, Shanghai, 200437 China

**Keywords:** Hepatocellular Carcinoma, Tumor microenvironment, Traditional Chinese medicine, Immune modulation, Therapy resistance, Combination therapy

## Abstract

Hepatocellular carcinoma (HCC) progression and therapeutic resistance are profoundly influenced by the dynamic interplay within the tumor microenvironment (TME). The HCC TME comprises a complex network of cellular components, including cancer-associated fibroblasts, tumor-associated macrophages, and infiltrating immune cells, alongside non-cellular factors such as extracellular matrix proteins, cytokines, and angiogenic mediators. These elements collectively promote immune evasion, stromal remodeling, and neovascularization, driving tumor aggressiveness and treatment resistance. Emerging evidence suggests that traditional Chinese medicine (TCM) may offer a promising strategy to reprogram the immunosuppressive HCC TME through multimodal mechanisms, such as immunomodulation to enhance anti-tumor immunity and deplete regulatory cell populations, stromal normalization to attenuate fibroblast activation and pathological matrix deposition, and anti-angiogenic effects to restrict tumor vascularization. Notably, TCM compounds exhibit synergistic potential when combined with conventional therapies, including immune checkpoint inhibitors, tyrosine kinase inhibitors, and cytotoxic regimens, potentially enhancing efficacy while mitigating adverse effects. However, key challenges persist, such as intratumoral heterogeneity, pharmacokinetic variability of herbal formulations, and the need for rigorous preclinical-to-clinical translation. Future investigations should prioritize systems-level dissection of TCM-mediated TME modulation using omics technologies, rational design of TCM-based combination therapies guided by mechanistic studies, and standardization of clinically translatable TCM regimens. This review synthesizes current understanding of TME-driven HCC pathogenesis and highlights the emerging paradigm of TCM as a complementary modality to recalibrate the tumor-immune-stroma axis for improved therapeutic outcomes.

## Introduction

Hepatocellular carcinoma (HCC) is a malignant tumours with high global morbidity and mortality worldwide. According to the World Health Organization (WHO) 2020 statistics, HCC ranks as sixth most common cancer worldwide, with over 900,000 new cases annually, and the third leading cause of cancer-related deaths, causing approximately 830,000 deaths per year [1]. The high prevalence of HCC is closely associated with chronic liver diseases, especially hepatitis B virus (HBV) and hepatitis C virus (HCV) infections, alcoholic liver disease, and non-alcoholic fatty liver disease (NAFLD). Furthermore, cirrhosis is a major risk factor for HCC development,present in about 80%−90% of patients atdiagnosis [2, 3]. Despite significant progress in HCC diagnosis and treatment, its prognosis remains poor. Early-stage patients can achieve better outcomes with surgical resection, liver transplantation, or local ablation. However, most patients are in the middle to late stage at the time of diagnosis, losing the opportunity for surgery. For patients with advanced HCC, systemic therapies (e.g., targeted therapy and immunotherapy) are the mainstay of treatment [4–6]. However, there are many limitations of existing treatments in clinical application: 1) surgical resection requires high liver function of patients, and the location of the tumour also affects the feasibility of surgery, and the recurrence rate is as high as 70% within five years after surgery; 2) although liver transplantation may be curative, strict Milanese criteria limit the proportion of patients suitable for transplantation to less than 30%; 3) the first-line targeted drugs, represented by sorafenib, have less than 20% effective rate and there is a general problem of drug resistance; 4) although immunotherapies such as PD-1 inhibitors have opened up new avenues, the effective rate is only 15%−20% when used alone, indicating that individual differences have a significant impact on the efficacy of treatment [7]. In summary, the high morbidity and mortality of HCC and the limitations of existing treatments urgently require us to explore new therapeutic strategies and targets.

Genomic instability and mutations in cancer cells are considered to be fundamental driving features during cancer progression [8]. Therefore, a great deal of research has been largely confined to the tumor cells themselves. However, tumors are not just a genetic disease, but a complex cellular ecosystem involving a wide range of non-cancerous cells and their complex interactions within the tumor. In 1889, Stephen Paget laid the foundation for the concept of the TME with his “seed and soil” hypothesis, which describes the tumor cell as a “seed” whose development and metastasis depend on the surrounding microenvironmental “soil”. The “seed and soil” hypothesis laid the foundation for the concept of TME [9]. Studies have shown that the occurrence, progression and recurrence of HCC are closely related to its microenvironment [10]. Therefore, targeting TME has evolved as a potential therapeutic strategy.

As a unique medical system of TCM, Chinese medicine has a history of development of thousands of years, and its theoretical framework and clinical practice system have been perfected after a long period of accumulation. In the field of malignant tumour prevention and treatment, TCM has become an indispensable part of the Chinese medical system [11]. Guided by its holistic philosophy and syndrome differentiation principles, TCM posits that HCC pathogenesis arises from multifactorial interactions, including vital qi deficiency, pathogenic factor invasion, and qi-blood stasis. Through personalized pattern differentiation, TCM adopts a systemic approach to HCC management. By implementing syndrome-based individualized regimens, TCM develops tailored therapeutic strategies that modulate the TME, suppress neoplastic proliferation, and enhance patients’ quality of life. TCM employs a wide range of therapeutic approaches, such as herbal monomers, bioactive extracts, classical prescriptions, standardized compound formulations, and acupuncture. Compared with conventional antitumor therapies, TCM preparations exhibit distinct advantages such as high accessibility, reproducible efficacy, and manageable adverse effects, positioning them as a pivotal research focus in integrative oncology [12]. Notably, the synergistic application of TCM with modern therapeutic strategies (including immune checkpoint blockade, molecular targeted therapy, and radiotherapy) presents significant synergistic and toxicity-reducing effects in the clinical management of HCC. For example, TCM can significantly inhibit tumour growth, reverse drug resistance, and attenuate the adverse effects of targeted therapy and chemotherapy/radiotherapy in antitumour therapy, thereby improving patients'quality of life [13, 14]. In recent years, molecular mechanism studies have revealed that TCM can dynamically regulate TME through a multi-component and multi-target mode of action, intervening in key biological processes such as tumour immune escape and angiogenesis [15]. This review systematically examines HCC microenvironment heterogeneity and its pathological evolution, analyzes TCM-mediated TME regulation mechanisms, and identifies bioactive TCM compounds with therapeutic potential. The study further explores integrative treatment networks combining TCM with modern therapies, providing theoretical foundations and translational insights to innovate precision treatment models for HCC management.

## Composition and dynamics of the HCC tumour microenvironment

TME plays a critical role in influencing tumor growth, metastasis, and response to therapy. The interactions between various cellular and noncellular components of the TME create a supportive ecological niche for cancer cells, promoting their survival, proliferation, and spread [16]. These components can either promote or inhibit tumor progression, depending on the nature of the interaction and the balance of pro- and anti-tumor signals.

### Cellular component

TME refers to the surrounding tissues and cell populations that enclose malignant tumor cells and consists of a wide variety of different tumor-infiltrating nonmalignant cells as well as extracellular matrix (ECM), the composition of which varies depending on the tumor type, and it is an important environment for the growth and metastasis of malignant tumors [17]. Key components of TME include 1) Cancer-Associated Fibroblasts (CAFs); 2) Tumor-Infiltrating Immune Cells (neutrophils, Macrophages, Dendritic Cells (DCs), Natural Killer Cells (NKs), Myeloid-Derived Suppressor Cells (MDSCs), and CD8^+^ T Cells, CD4^+^ T Cells, Tregs, and B Cells); 3) ECM; 4) Blood Vessels; and 5) Cytokines. These cellular components produce non-cellular components of the tumor stroma, including different growth factors, cytokines, chemokines, proteins, protein hydrolases and their inhibitors, and inflammatory cytokines, and interact with HCC cells through a complex communication network [18–20]. The interactions between tumor cells and their microenvironment are dynamic and bidirectional, and the main body of communication mechanisms is divided into 1) contact-dependent mechanisms of cell-to-cell contact or cell-to-ECM contact, and 2) non-contact-dependent mechanisms of soluble molecules (growth factors, chemokines, cytokines, and subcellular structures, including microvesicles and exosomes) [21]. However, tumor heterogeneity is caused by various signaling pathways/crosstalk present in the network of communicating cancer cells [22]. Therefore, a comprehensive analysis of the interactions between HCC cells and their TME is essential to understand the different underlying mechanisms of HCC growth and metastasis.

#### Role of cancer-associated fibroblasts (CAFs) in HCC progression

CAFs are the predominant mesenchymal cell population in the HCC microenvironment, accounting for approximately 50%−70% of the total number of TME cells. These cells play a pivotal role in the genesis, metastasis and treatment resistance of HCC through dynamic remodelling of the ECM structure, secretion of growth factors and modulation of immune response. Recent studies have revealed that the functional diversity and high heterogeneity of CAFs make them an important breakthrough for targeted therapy of HCC.

In HCC, activation of CAFs is a complex process involving multiple cell types and signaling pathways. It mainly originates from the transdifferentiation of hepatic stellate cells (HSCs): resting HSCs are stimulated by cytokines, such as TGF-β and PDGF, in TME, and transformed into activated myofibroblast-like CAFs, a process that is closely related to the background of hepatic fibrosis/cirrhosis, and is accompanied by fibrotic milieu in more than 90% of HCC cases [23–25]. In addition, CAFs can be differentiated by epithelial-mesenchymal transition (EMT), endothelial-mesenchymal transition (EndMT), bone marrow-derived mesenchymal stem cells (MSCs), adipocytes, and pericytes from [26, 27]. This multidimensional activation mechanism leads to a significant heterogeneity of CAFs in the TME, which lays the foundation for their functional diversity.

CAFs synergistically drive the malignant progression of HCC through a multi-pathway interaction network. First, CAFs directly promote tumour proliferation and metastasis by secreting growth factors (e.g., CXCL11, CCL5): CAF-derived CCL5 enhances HCC metastasis by triggering the HIF1α/ZEB1 axis, while CXCL11 directly promotes HCC cell proliferation [28, 29]. Secondly, CAFs indirectly support tumour progression by remodelling the immune microenvironment: on the one hand, it suppresses both CD8^+^ T cell infiltration and function by recruiting MDSCs and Tregs; on the other hand, TGF-β released by CAFs induces the polarisation of M2-type macrophages and promotes the depletion of T cells by delivering molecules such as miR-1228-3p via exosomes to form a multi-layered immune escape barrier [30–32]. In addition, CAFs provide the basis for tumour spread by driving angiogenesis and metastasis: by secreting pro-angiogenic factors (e.g. VEGF, PDGF, etc.) and remodelling the ECM, they provide tumours with nutrients and oxygen to support their growth and spread [19, 33, 34]. More critically, CAFs mediate therapeutic resistance through chemoresistance and targeted therapeutic barriers: CAFs deliver molecules such as miR-1228-3p via exosomes to induce resistance of HCC cells to drugs such as sorafenib; at the same time, activation status of CAFs (e.g., high expression of FAP) correlates with HCC postoperative recurrence and may be a therapeutic target [32, 35, 36]. Together, these mechanisms constitute the core network of CAFs promoting HCC progression, highlighting their central regulatory position in the TME.

The heterogeneity of CAFs is an important source of their functional complexity. In recent years, the application of single-cell sequencing technology has revealed the highly heterogeneous nature of CAFs. Based on their functional characteristics, CAFs can be classified into pro-tumour (pCAFs) and anti-tumour (rCAFs). pCAFs are characterized by high expression of α-SMA and FAP, exhibiting potent tumor-promoting and immunosuppressive properties. In contrast, rCAFs demonstrate elevated expression of CD146 and PDGFRβ, which may mediate tumor-suppressive effects [37]. This molecular heterogeneity of CAF subpopulations presents novel therapeutic targets for precision medicine approaches in HCC treatment.

#### The dual role of tumour-associated macrophages (TAMs) as pro- and anti-tumour agents

As the highest proportion of immune cell population (30%−50%) in the HCC microenvironment, the functional polarisation of TAMs is dynamically regulated by microenvironmental signals and plays a dual role in the pro- and anti-tumour processes.

In the early stage of HCC progression, peripheral blood mononuclear cells are recruited to the HCC microenvironment via chemokines such as CCL2 and CSF-1, and subsequently differentiate into M1- or M2-type TAMs with very different functions [38, 39]. Among them, M1-type polarisation is driven by activation of IFN-γ and LPS (Lipopolysaccharide), which confers pro-inflammatory and anti-tumour activities to TAMs, whereas IL-4, IL-10, and IL-13 induce M2-type polarisation, forming a pro-carcinogenic microenvironment with immune-suppressive, pro-angiogenic and metastasis-promoting effects [40, 41]. Notably, the characteristic hypoxic features of HCC further enhanced M2-type polarisation through the HIF-1α-VEGF pathway, leading to a significantly higher percentage of M2-type TAMs in TME, an imbalanced state that is closely associated with tumour immune escape and poor prognosis [42, 43].

The immunosuppressive function of M2-type TAMs is achieved through a multidimensional mechanism: on the one hand, its highly expressed PD-L1 on the surface binds to T cell PD-1 and inhibits T cell activity, thus helping the tumour cells to escape from immunosurveillance; on the other hand, it facilitates immune escape through the secretion of immunosuppressive cytokines (e.g., IL-10, TGF-β) and the inhibition of T cell function [44–46]. In addition, TAMs promote angiogenesis and extracellular matrix remodelling through the secretion of vascular endothelial growth factor (VEGF) and matrix metalloproteinases (MMPs), creating favourable conditions for tumour metastasis [44, 47]. Therefore, inhibiting M2 polarisation and inducing repolarisation towards M1 may help to stimulate anti-tumour specific immune responses and reduce tumour metastasis [48].

#### Functions of tumour infiltrating lymphocytes (TILs) and their significance in immunotherapy

T cells are an important component of the unique immune microenvironment of HCC, influencing tumour growth and therapeutic response by directly or indirectly participating in the immune response. Depending on their intrinsic structure and potential functions, T cells are mainly classified into two different subtypes: CD8^+^ T cells and CD4^+^ T cells. CD8^+^ T cells are the main effector cells of the anti-tumour immune response. Initial CD8^+^ T cells differentiate into CD8^+^ cytotoxic T lymphocytes (CTLs) after specifically recognising antigenic peptides presented by antigen-presenting cells (APCs) via MHC-I [49]. Activated CD8^+^ T cells play a key role in anti-tumour immunity by transporting to the TME secreting factors such as perforin, TNF-α and FASL to exert cytotoxic effects performing effector functions to induce target cell death [50]. Therefore, TCM can exert anti-tumour efficacy by targeting and promoting CD8^+^ T cell activation. Regulatory T cells (Tregs) are a special subset of CD4^+^ T cells known for their immunosuppressive function. Mechanisms that contribute to Treg's ability to achieve their immunosuppressive capacity in the TME include:(1) direct lysis of effector T cells through the release of granzyme B and perforin, (2) induction of apoptosis of effector T cells through deprivation of IL-2 by high-affinity CD25, (3) suppression of immune responses through the induction of tolerogenic dendritic cells, and (4) release of inhibitory cytokines, including TGF-β, IL-10, IL-35, and prostaglandin E2 to modulate effector cell immune responses [51, 52]. In addition, Tregs inhibit the tumour-killing activity of CD8^+^ T cells by secreting inhibitory cytokines, inhibiting cell-to-cell contact and interfering with effector cell metabolism. Another CD4^+^ T cell subset, Th1, supports the immune response mainly by promoting CD8^+^ T cell activation and stimulating anti-tumour immune responses [53]. Given the key role played by various T-cell subpopulations in the TME, therapeutic strategies targeting T-cells are widely regarded as a highly promising new approach to tumour treatment.

In addition, cancer cells are able to activate multiple immune checkpoint pathways that contribute to tumour immune escape, with programmed death protein 1 (PD-1) and programmed cell death ligand (PD-L1) considered to be the main immune checkpoint molecules [54]. PD-1 is a common immunosuppressive factor on the surface of T cells, and PD-L1 is overexpressed on the surface of malignant tumour cells, whereas the interaction between PD-1 and PD-L1 leads to therapeutic failure by inhibiting the activity of effector T cells and enhancing the function of immunosuppressive Tregs and ultimately inducing immune escape [55]. Therefore, TCM can act as a PD-1/PDL-1 checkpoint inhibitor, which can block the PD1-PD-L1 axis interaction and reverse the inhibition of T cells, thus destroying HCC cells and exerting anti-tumour effects [56].

#### Role of other immune cells

MDSC are known for their remarkable immunosuppressive properties and are able to efficiently modulate a wide range of immune cell-mediated immune responses, and these cells can be activated by tumour-derived factors in the TME. MDSC can be subdivided into two major phenotypes: polymorphonuclear MDSC (PMN-MDSC) and mononuclear MDSC (M-MDSC) [57]. Notably, compared to MDSC in peripheral lymphoid organs, M-MDSC and PMN-MDSC located in the TME exhibited a more enhanced inhibitory phenotype, which was attributed to a significant increase in the expression levels of their inhibitory molecules in the TME. Specifically, PMN-MDSC mediates immunosuppressive effects through the release of reactive oxygen species (ROS), arginase 1 (arg-1), and prostaglandin E2 (PGE2), whereas M-MDSC utilises nitric oxide, immunosuppressive cytokines (e.g., IL-10 and TGF-β), and immunomodulatory molecules (e.g., programmed death ligand 1, PD-L1) to achieve its immunosuppressive function [57]. Current research trends suggest that TCM may exert its effects through multiple pathways, including removal of MDSC and inhibition of their recruitment and activity at the tumour site.

### Non-cellular component

#### Remodelling of the extracellular matrix (ECM) in relation to HCC metastasis

The ECM is a complex network of macromolecules, including collagen, fibronectin, elastin, proteoglycans and glycosaminoglycans, that provide structural and biochemical support to surrounding cells [58]. In addition to its structural role, the ECM plays an active role in regulating cell behaviour and influencing cell migration, differentiation and survival. HCC is the most common type of liver cancer. Chronic liver diseases such as cirrhosis lead to the accumulation of ECM proteins, resulting in fibrosis. The fibrotic tissue creates a rigid and supportive scaffolding for the cancer cells, which promotes the growth and survival of the HCC cells. ECM remodelling is usually characterised by the activation of fibroblasts and myofibroblast activation, which produce increased ECM components (e.g., collagen) that can promote a more invasive environment for cancer cells. The ECM not only supports tumour growth, but also plays a critical role in the ability of cancer cells to invade surrounding tissues and metastasise to distant organs. Changes in the composition of the ECM can promote the degradation of ECM components, with MMPs being the enzymes that MMP are enzymes that degrade ECM proteins, thereby making it easier for cancer cells to invade surrounding tissues and blood vessels [58]. In addition, the ECM can regulate the formation of new blood vessels or angiogenesis, which is essential for providing nutrients to growing tumours. Tumour cells can secrete ECM-modifying enzymes that stimulate angiogenesis, thereby promoting the metastatic ability of tumours. In summary, the ECM plays a key role in the progression of HCC by providing structural support to tumours, promoting invasion and metastasis, and interacting with other components of the TME.

#### The regulatory role of cytokines and chemokines in the microenvironment

In the malignant progression of HCC, cytokines and chemokines play a central role by dynamically regulating the immunosuppressive properties and pro-metastatic ability of TME. Chemokines represented by CXCL10 and CCL17 recruit Tregs and TAMs to infiltrate into the TME through ligand-receptor specific binding (e.g., CCL17-CCR4 axis), where TAMs differentiate into an M2-type phenotype under the polarising effect of IL-6 and TGF-β, and inhibit cytotoxicity function of CD8^+^ T cells by secreting PD-L1 and IL-10, and forming multiple immune escape barriers [59–62]. Meanwhile, CAFs and HSCs activate pro-fibrotic and angiogenic pathways by secreting TGF-β and VEGF, driving fibrotic remodelling of the ECM and aberrant vasculogenesis, and providing biochemical scaffolds for HCC cell proliferation and invasion [63–65]. Notably, chemokines (e.g. CXCL1) in the context of chronic inflammation maintain a pro-inflammatory milieu through NF-κB signaling, inducing EMT and accumulating mutations that transform HCC from a local lesion to systemic metastasis [65, 66].

The functions of chemokines in TME show remarkable duality: while CCL22 inhibits anti-tumour immunity by recruiting Tregs through a CCR4-dependent pathway, CXCL10 enhances immunosurveillance by recruiting CXCR3^+^ effector T-cells, a paradoxical effect that may stem from the spatial and temporal heterogeneity of the cytokine concentration gradient and the expression profile of the receptor within the microenvironment [67]. In addition, there is cross-regulation of chemokines with immune checkpoint molecules, for example, CCL2 synergistically up-regulates PD-L1 expression, suggesting that targeting chemokine networks (e.g., CCR4 inhibitors or CXCL10 agonists) may be a potential strategy to enhance immunotherapeutic response [68, 69]. From a clinical translational perspective, the expression levels of specific chemokines have prognostic indicative value: high expression of CCL16 is significantly associated with shorter overall survival in HCC patients, whereas elevated levels of CXCL10 are indicative of active T-cell infiltration and potential benefit from immunotherapy [60, 70]. Based on this, intervention strategies present a bidirectional approach—inhibition of pro-tumour factors (e.g. IL-6 monoclonal antibody) or activation of anti-tumour signals (e.g. upregulation of CXCL10 by HDAC3 inhibitors) can both remodel the TME ecology, whereas targeting the TGF-β-mediated fibrotic process (e.g. Galunisertib) also reduces microenvironmental stiffness to reverse mechanical stress-driven YAP/TAZ activation and achieve multidimensional anti-tumour effects [59, 60, 71].

In summary, cytokines and chemokines in the TME of HCC regulate immunosuppression, tumour proliferation and metastasis through a complex network, and their dual roles (pro- or anti-tumour) depend on the specific molecule type and microenvironmental physicochemical properties. Intervention strategies targeting these molecules (e.g., blocking pro-tumour factors or enhancing anti-tumour immune recruitment) provide new directions for HCC therapy.

#### Effect of angiogenic factors on vascular neogenesis in HCC

The formation of the neovascular system plays an important role in the development and metastasis of HCC as it allows the tumour to grow beyond the diffusion limits of oxygen and nutrients. With rapid tumour growth, hypoxia occurs within solid tumours due to high interstitial pressure and the distance between tumour cells and adjacent capillaries, and hypoxic conditions are central to shaping TME. This hypoxic condition triggers the activation of HIF, which induces the expression of pro-angiogenic factors such as VEGF [72, 73]. Most in vitro and in vivo HCC models investigating hypoxia-mediated mechanisms in HCC have focused on the upregulation of hypoxia-inducible factor-1α (HIF-1α), a protein that induces the expression of pro-angiogenic factors, including VEGF, platelet-derived growth factor (PDGF), fibroblast growth factor (FGF), and angiopoietin, which can promote HCC angiogenesis in HCC tumours, thus creating a positive pathogenic feedback loop [74–76]. Stromal cells associated with tumour tissue play a key role in HCC angiogenesis [77]. Activated haematopoietic stem cells (a-HSC) can secrete pro-angiogenic factors or cytokines, including VEGFA, PDGFB and Angs, to promote HCC angiogenesis [78, 79]. Crosstalk between HCC cells and HSC also affects angiogenesis, e.g., PDGF-BB and SHh secreted by HCC cells activate HSC, which in turn promotes angiogenesis [80, 81]. In addition, M2-like TAM promotes angiogenesis in HCC by secreting pro-angiogenic factors or cytokines [82]. Interestingly, recent studies have also highlighted the role of exosomes (small vesicles released by cells) and microRNAs (miRNAs) in the communication between tumour cells and the surrounding microenvironment. Exosomes can transfer signaling molecules, such as growth factors and miRNAs, between cells, thereby influencing the behaviour of stromal cells, immune cells and other components of the TME. For example, the HCC cell-derived exosome miR-21 converts HSC to CAF by inhibiting PTEN and activating PDK1/AKT, which in turn promotes angiogenesis by secreting VEGF, MMP2, MMP9, bFGF, and TGF-β [83]. LncRNA cox-2, miR-140 and CSF-1 promote macrophage polarisation towards the M2 subtype, which secretes IL-10, TGF-β and PGE2, thereby promoting angiogenesis in HCC [84–86]. This leads to the formation of new blood vessels that provide the tumour with the oxygen and nutrients it needs for further growth, and also promotes the metastatic spread of HCC.

### Role of TME in HCC progression, metastasis and treatment resistance

#### Mechanisms of TME-mediated immune escape

Immunosuppression within the TME is a key factor in the interaction between tumour cells and surrounding stromal components that allows tumour progression by evading the body's immune surveillance. In HCC, immunosuppression within the TME is predominantly infiltrated by a variety of immune cells, such as macrophages, DCs, T cells and MDSCs. These immune cells can either promote or inhibit tumour growth, depending on their phenotype and the signals they receive from the tumour and the surrounding environment. TAMs are one of the most abundant immune cell types in the HCC microenvironment. TAMs can have either pro- or anti-tumour effects, depending on their polarisation status. M2 macrophages are usually associated with immunosuppressive and pro-angiogenic functions and usually predominate in HCC[87]. These macrophages secrete pro-tumour factors such as VEGF, IL-10 and TGF-β. These cytokines contribute to tumour growth, angiogenesis and suppression of anti-tumour immune responses. Tregs accumulate in the HCC microenvironment and produce immunosuppressive factors (e.g. TGF-β) that inhibit effector T cell activity and promote immune evasion [88–90]. This immunosuppression is a significant barrier to successful immunotherapy for HCC. MDSCs inhibit T-cell function by secreting ROS, arginine-1, and other immunosuppressive molecules, thereby promoting immune evasion [87]. Different types of immune cells in the TME play multiple roles such as immune surveillance, immune response and immune escape locally in the tumour, and their numbers, functional sta.

#### The role of TME in targeted therapy resistance

In the mechanism of targeted therapy resistance in HCC, TME forms a complex defence network through multidimensional dynamic regulation. The core feature of TME is manifested as the synergistic action of immunosuppressive cells and signaling molecules: TAMs are polarized to the M2 phenotype driven by IL-10 and TGF-β, which directly inhibit the function of CD8^+^ T cells through the secretion of PD-L1 and adenosine, and at the same time, induce the initiation of the tumour cells'autophagy pathway to escape killing by sorafenib [91–93]; CAFs, on the other hand, counteract the inhibitory effect of targeted drugs on tumour cell proliferation through the continuous activation of the PI3K/AKT pathway by paracrine hepatocyte growth factor (HGF) and VEGF [31, 63]. Notably, resistance is further exacerbated by the physical barrier properties of the TME-ECM components (e.g. collagen fibres) restrict drug penetration through a mechanical barrier, resulting in short retention times and uneven distribution of targeted drugs in the TME [94]. In addition, ECM remodelling activates the YAP/TAZ pathway through mechanical stress, further promoting tumour survival and drug resistance [58]. The maintenance of this resistance ecological niche also relies on CXCL12 secreted by CAFs, which recruits immunosuppressive cells and establishes an immune-immunity microenvironment in a CXCR4 receptor-dependent manner [95]. Even more challenging, targeted therapy itself may trigger compensatory remodelling of the TME: e.g. activation of CAFs or infiltration of immunosuppressive cells, creating secondary drug resistance [96, 97]. This dynamic remodelling allows the tumour to escape therapeutic pressure, leading to relapse. This multilevel dynamic interplay suggests that breaking through resistance to HCC targeted therapies requires the development of synergistic intervention strategies that target the spatiotemporal heterogeneity of the TME.

#### The role of TME in chemotherapy resistance

TME in HCC mediates chemoresistance through multilevel synergistic effects, and its core mechanisms encompass the dynamic interplay of immunosuppression, mesenchymal remodelling, and physical barriers. The immunosuppressive properties of TME are dominated by TAMs-M2-type TAMs inhibit the cytotoxicity of CD8^+^ T cells through the secretion of IL-10 and TGF-β, while enhancing the anti-apoptotic capacity of tumour cells, leading to a reduction in the killing efficiency of chemotherapeutic agents. In addition, Tregs further consolidated the immune escape network through CTLA-4-dependent depletion of effector T cells. Notably, the mesenchymal components of TME amplify the drug resistance effect through bidirectional interactions: CAFs induce the expression of multidrug-resistant proteins through the secretion of cytokines HGF and FGF; whereas aberrantly functioning tumour vascular endothelial cells (TECs) promote self-renewal of tumour stem cells through factors such as VEGF to form a subpopulation of cells with high drug resistance subpopulation of cells with high drug resistance potential [98]. In the physical barrier, collagen and fibronectin in the ECM also activate drug-resistant pathways within tumour cells through integrin signaling. The establishment of this multidimensional resistance network suggests that targeting the spatiotemporal heterogeneity of TME (e.g., combined inhibition of CAFs secretion profiles with ECM mechanosignaling) may be a key breakthrough point for reversing chemoresistance in HCC.

**Fig. 1:**
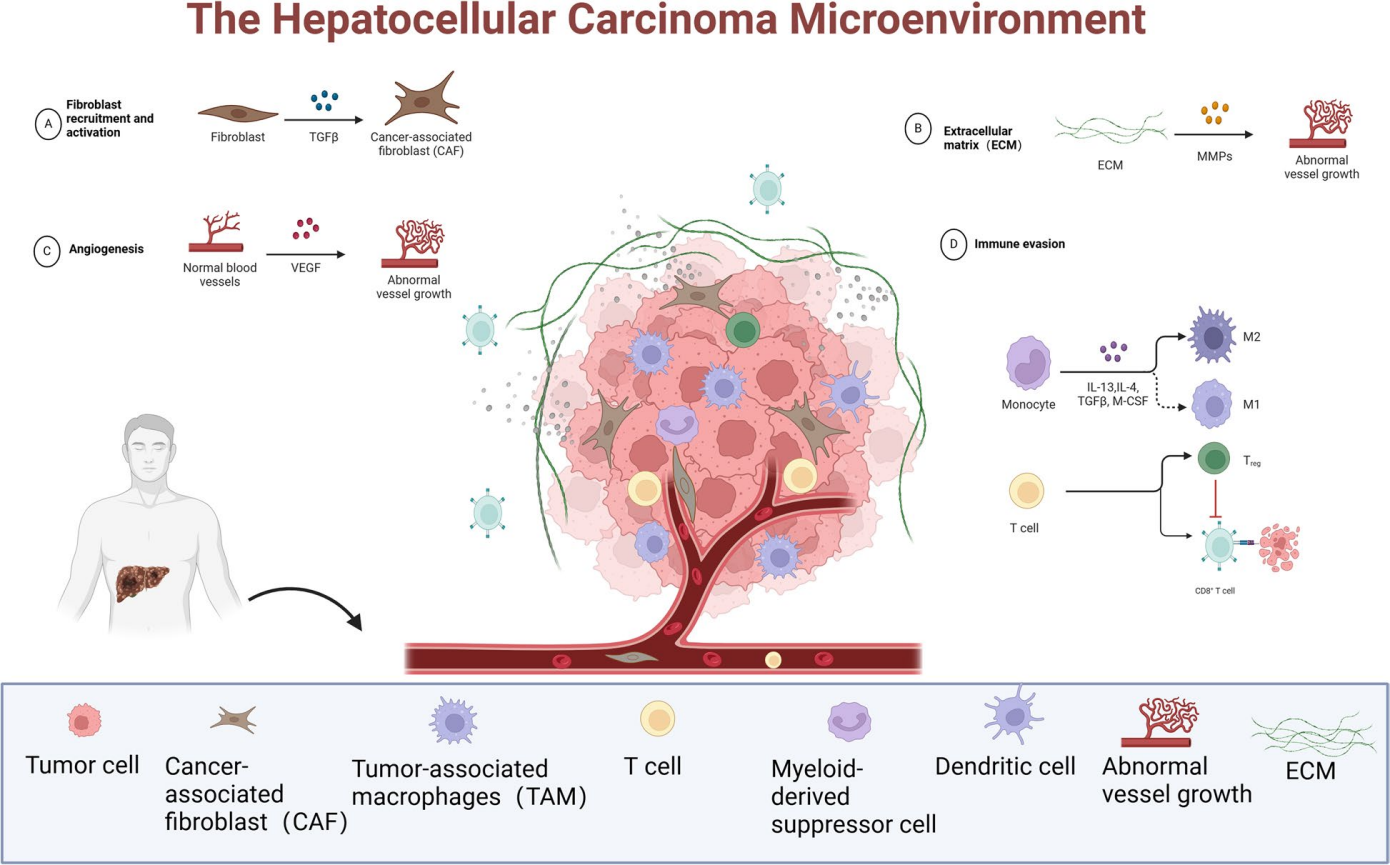
Overview of the hepatocellular carcinoma microenvironment. **A** In the TME, fibroblasts can differentiate into CAFs under the stimulation of various growth factors and cytokines, especially transforming growth factor-β (TGF-β). **B** When the ECM is degraded by proteases (e.g. MMPs), growth factors are released to promote angiogenesis. **C** In the TME, normal blood vessels undergo abnormal angiogenesis in response to VEGF. **D** Macrophages in the TME are polarized to tumor-promoting M2-type macrophages induced by cytokines such as IL-13, IL-4, TGF-β, and M-CSF; T-cell activity, in conjunction with other cells in the TME, indirectly promotes the recruitment and expansion of Tregs

In summary, the HCC microenvironment is influenced by a variety of factors, each of which contributes to the creation of a supportive ecological niche for HCC cells that promotes tumourigenesis, progression and metastasis. The overview of the HCC microenvironment is displayed in Fig. 1.

## Mechanisms of TCM in Modulating HCC microenvironment

The molecular pathogenesis of HCC is highly complex, involving intricate interactions within the TME. TCM has emerged as a compelling therapeutic option due to its multi-level, multi-targeted, and coordinated interventions. TCM offers a holistic approach, addressing various components of the TME simultaneously, which is crucial for effective HCC management. To date, numerous TCM extracts have demonstrated significant potential in modulating the HCC microenvironment through several key mechanisms. Immune Cell Modulation: TCM compounds have been shown to enhance the activity of immune cells such as NK cells, CTLs, and DCs. This immunomodulatory effect helps in promoting anti-tumor immunity and reducing immune evasion by HCC cells. Targeting CAFs: CAFs play a pivotal role in promoting tumor progression and resistance to therapy. TCM extracts can inhibit the activation and function of CAFs, thereby reducing their pro-tumorigenic effects and improving the efficacy of conventional therapies. ECM Remodeling: The ECM in HCC is often dysregulated, contributing to tumor growth, invasion, and metastasis. TCM has been found to modulate ECM components and enzymes, such as MMPs, thereby inhibiting tumor invasion and metastasis. Angiogenesis Inhibition: Angiogenesis is critical for tumor growth and metastasis. TCM compounds can block angiogenesis by inhibiting VEGF signaling and other angiogenic pathways, thus starving the tumor of necessary nutrients and oxygen.

**Fig. 2:**
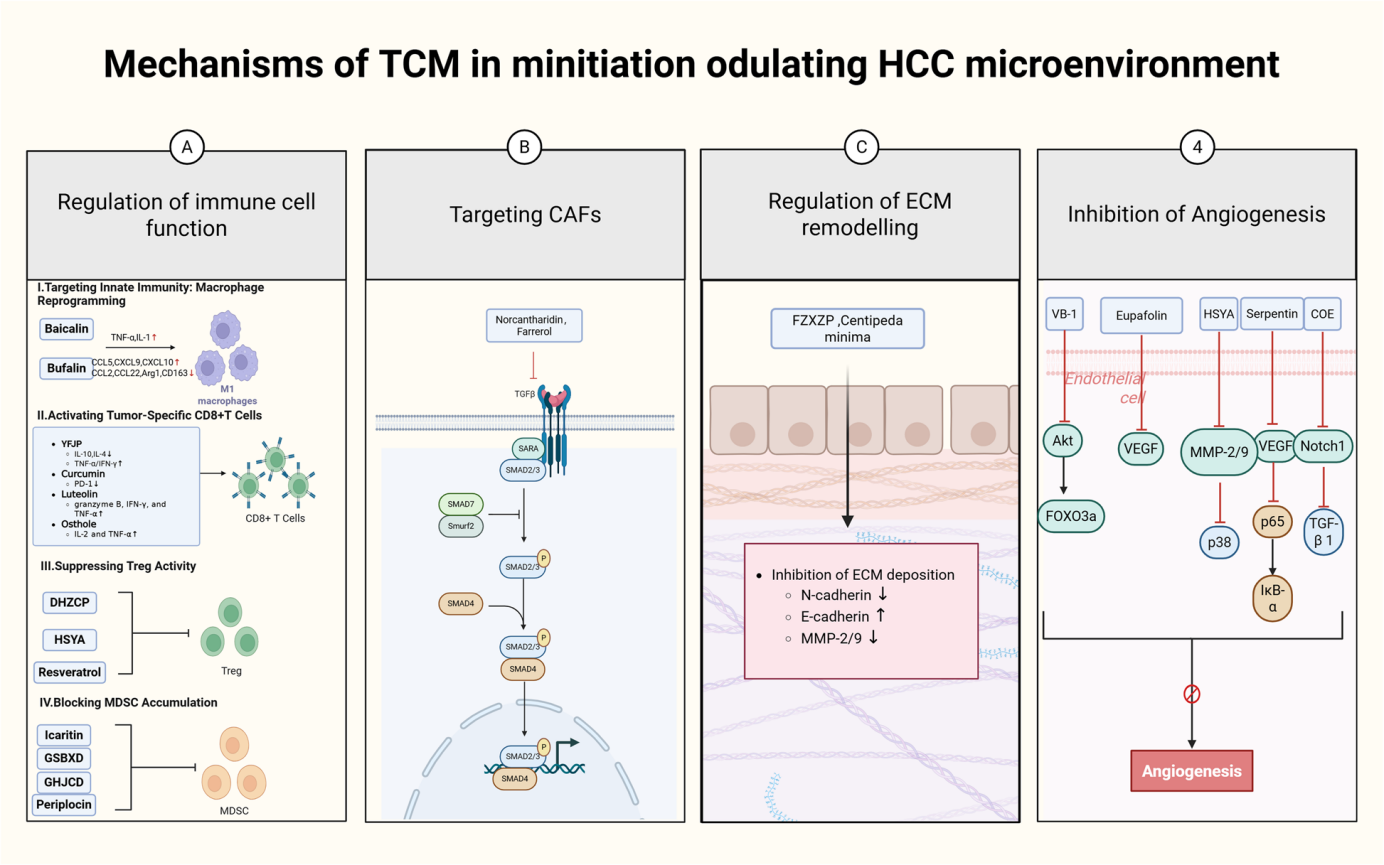
Mechanisms by which TCM regulates the HCC microenvironment. **A** In TME, TCM can promote anti-HCC therapy by regulating the function of immune cells. **B** By targeting CAFs, TCM can inhibit their tumour-promoting function and reverse the state of immunosuppression in TME. **C** By regulating the remodelling of the ECM, TCM can inhibit HCC metastasis. **D** By inhibiting angiogenesis, TCM can inhibit the growth and metastasis of HCC

These mechanisms collectively highlight the potential of TCM in reshaping the HCC microenvironment, making it less conducive for tumor growth and more responsive to therapeutic interventions. The multi-faceted approach of TCM aligns well with the complexity of HCC pathogenesis, offering a promising avenue for developing novel and effective treatment strategies (as shown in Fig. 2).

### Regulation of immune cell function

Immune cells play a key regulatory role in the TME of HCC, and the dynamic balance of their functional phenotypes profoundly affects tumour development, metastasis and treatment outcome. Studies have shown that immune cells in the TME have significant dual-functional characteristics: on the one hand, they exert anti-tumour effects through antigen presentation and cytotoxicity, and on the other hand, they can mediate tumour immune escape through immune checkpoint activation and inhibitory cytokine secretion. Notably, increasing evidence reveals that TCM can reshape the immune homeostasis of TME through multi-targeted modulation, including the enhancement of anti-tumour immune responses and the inhibition of immunosuppressive cell functions, providing a new strategy for precision immunotherapy in HCC [99]. The immunomodulatory effects of TCM are systematically summarized in Table 1.

**Table 1:** TCM regulates immune cell function

Mechanism	Compound/single TCM/extract/formula	Origin	Mechanism	Ref
TAM	Baicalin	Scutellaria baicalensis Georgi	converts M2-TAMs to M1 phenotypes (CD86^+^↑, CD206^+^↓) within 48 h by inducing TRAF2 autophagic degradation	[100]
	Bufalin	Chansu	mediates a shift in NF-κB dimerization from immunosuppressive p50 homodimers to immunostimulatory p65-p50 heterodimers	[101]
T Cell	Yangyin Fuzheng Jiedu Prescription (YFJP)	/	reduces PD-1/TIM-3⁺ exhausted CD8⁺ T cells by suppressing IL-10/IL-4 and enhancing TNF-α/IFN-γ via inhibiting PI3K-Akt signaling	[102, 103]
	Curcumin	Turmeric	downregulates CD8^+^ T cell PD-1 expression and inhibits TGF-β1/Smad signaling via suppressing thrombin-P300-mediated histone acetylation	[104]
	Luteolin	/	restores the cytotoxic activity of tumor-infiltrating CD8^+^ T cells by upregulating granzyme B, IFN-γ, and TNF-α expression	[105]
	Osthole	Fructus Cnidii	elevates tumor-infiltrating CD4 +/CD8^+^ T cell ratio and serum IL-2/TNF-α levels while reducing splenic Tregs	[106]
	Cryptotanshinone (CT)	Salvia miltiorrhiza	synergizes with anti-PD-L1 therapy via TLR7/MyD88/NF-κB signaling, enhancing dendritic cell activation and CD8 + T cell effector/memory responses to establish long-term anti-tumor immunity	[107]
	Dahuang Zhechong pill (DHZCP)	/	promotes Th1 cells and IFN-γ secretion to activate CD8^+^ T cells; inhibits Treg production and function	[108]
	Hydroxyl safflower yellow A (HSYA)	Carthamus tinctorius L	alleviates immunosuppression by reducing splenic Foxp3^+^Treg proportion and downregulating Foxp3 and Rorγt mRNA expression	[109]
	Resveratrol	White hellebore	reduces CD8^+^CD122^+^ Treg frequency and increases IFN-γ^+^ CD8^+^ T cells, enhancing anti-tumor immunity	[110]
MDSC	Icaritin	Epimedium spp.	suppresses tumor progression by attenuating splenic EMH, leading to reduced MDSC generation/activation and enhanced CD8^+^ T cell cytotoxicity	[111]
	Gan Shui Ban Xia Tang (GSBXD)	/	inhibits MDSC proliferation (via AKT/STAT3/ERK suppression) and pro-inflammatory cytokines (IL-1β, IFN-γ)	[112]
	Gehua Jiecheng Decoction (GHJCD)	/	downregulates Tregs, TAMs, MDSCs; suppresses IL-6, IL-10, TNF-α, CCL-2, CD31, VEGF via Wnt/β-catenin and NF-κB inhibition	[113]
	Periplocin	Periplocae Cortex	blocks AKT/NF-κB to suppress MDSC accumulation via inhibiting CXCL1/CXCL3 chemotaxis in HCC	[114]

#### Enhancement of anti-tumour immune response

##### Targeting Innate Immunity: Macrophage Reprogramming

The polarization switch between M1 and M2 macrophage phenotypes serves as a critical molecular regulator of immune surveillance in the TME. TCM compounds precisely reprogram macrophage polarization through key signaling pathways: Baicalin converts M2-TAMs to M1 phenotypes within 48 hours by upregulating CD86^+^ and downregulating CD206^+^ markers. Mechanistically, it induces autophagic degradation of TRAF2, activating the RelB/p52 pathway to drive TNF-α/IL-12 production. Functional validation confirms that autophagy/RelB inhibition abolishes TAM repolarization [100]. Similarly, Bufalin shifts NF-κB dimerization from immunosuppressive p50 homodimers to immunostimulatory p65-p50 heterodimers, promoting Th1 chemokines (CCL5, CXCL9/10) while suppressing M2 markers (CCL2, CCL22, Arg1, CD163) [101].

##### Activating Adaptive Immunity: Tumor-Specific CD8^+^T Cells

Beyond innate immunity, TCM significantly enhances adaptive immune responses by activating tumor-specific CD8^+^ T cells and NK cells. These effector cells are central to tumor immunosurveillance, and their functional status directly determines immunoclearance efficacy. TCM enhances tumor antigen-specific CD8^+^ T cell responses through three synergistic mechanisms: 1) Reversal of T Cell Exhaustion. Yangyin Fuzheng Jiedu Prescription (YFJP) significantly reduces the population of PD-1/TIM-3 exhausted CD8^+^ T cells in both tumor microenvironments and peripheral tissues. This is achieved through the suppression of IL-10/IL-4 secretion and the enhancement of TNF-α/IFN-γ production, mediated by the inhibition of the PI3K-Akt signaling pathway [102, 103]. Additionally, Curcuma longa extract (curcumin) downregulates PD-1 expression on CD8^+^ T cells and epigenetically inhibits the TGF-β1/Smad2/3 signaling axis by attenuating thrombin-P300-mediated histone acetylation [104].2) Enhancement of Effector Function and Infiltration. Luteolin has been demonstrated to restore the cytotoxic activity of tumor-infiltrating CD8^+^ T cells in H22 tumor models. This is evidenced by the upregulation of granzyme B, IFN-γ, and TNF-α expression in CD8^+^ T cells isolated from blood, spleen, and tumor tissues [105]. Furthermore, osthole increases the tumor-infiltrating CD4^+^/CD8^+^ T cell ratio and elevates serum levels of IL-2 and TNF-α, while concurrently reducing the population of splenic regulatory Tregs [106]. 3) Synergistic Effects with Immunotherapy. Cryptotanshinone (CT) exhibits a synergistic effect with anti-PD-L1 therapy by enhancing dendritic cell activation and promoting CD8^+^T cell effector/memory responses. This is mediated through the TLR7/MyD88/NF-κB signaling pathway, which contributes to the establishment of long-term anti-tumor immunity [107]. Similarly, luteolin amplifies the efficacy of anti-PD-1 therapy by expanding functional CD8^+^ T cell clones [105]. Collectively, these TCM-based interventions address CD8^+^T cell dysfunction within the immunosuppressive TME, providing a robust scientific rationale for the integration of TCM with combination immunotherapy strategies. This multi-faceted approach highlights the potential of TCM in enhancing anti-tumor immune responses and overcoming therapeutic resistance.

#### Inhibition of immunosuppressive cell function

##### Suppressing treg activity

The over-infiltration of immunosuppressive cells, such as Tregs and MDSCs, within the HCC microenvironment is a critical driver of immunotherapy resistance. TCM addresses this challenge by targeting key signaling pathways to suppress the differentiation and functional activity of these immunosuppressive cells. For Tregs，Dahuang Zhechong Pill (DHZCP) has been shown to enhance the population of Th1 cells in peripheral blood and spleen, thereby promoting the secretion of IFN-γ, which in turn activates CD8^+^ T cells. Concurrently, DHZCP inhibits the production and suppressive function of Tregs, thereby mitigating their immunosuppressive effects [108]. Hydroxyl safflower yellow A (HSYA) significantly reduces the proportion of FOXP3^+^ Tregs in the spleen and downregulates the expression of Foxp3 and Rorγt mRNA. This alleviates immunosuppression without inducing adverse effects such as weight loss, highlighting its therapeutic potential [109]. Additionally, resveratrol decreases the frequency of CD8^+^CD122^+^ Tregs in both tumor tissues and lymphoid organs, while simultaneously elevating the population of IFN-γ-producing CD8^+^ T cells, thereby enhancing anti-tumor immunity [110]. 

##### Blocking MDSC accumulation

In addition to its effects on Tregs, TCM effectively disrupts the accumulation and function of MDSCs through targeted pathway-specific interventions. Icaritin has been shown to suppress tumor progression by attenuating splenic extramedullary hematopoiesis (EMH), thereby reducing the generation and activation of MDSCs while simultaneously enhancing the cytotoxic activity of CD8^+^ T cells [111]. Gan Shui Ban Xia Tang (GSBXD) inhibits MDSC proliferation by downregulating the phosphorylation of AKT, STAT3, and ERK signaling pathways, as well as suppressing the production of IL-1β and IFN-γ. Concurrently, GSBXD increases the population of NK cells, further bolstering anti-tumor immunity [112]. Gehua Jiecheng Decoction (GHJCD) exerts a multifaceted immunomodulatory effect by downregulating Tregs, TAMs, and MDSCs within the HCC microenvironment. Additionally, GHJCD suppresses the secretion of inflammatory cytokines (e.g., IL-6, IL-10, TNF-α, and CCL-2) and inhibits angiogenesis markers (e.g., CD31 and VEGF) through modulation of the Wnt/β-catenin and NF-κB signaling pathways [113]. Notably, periplocin, a bioactive compound derived from Periploca sepium, suppresses MDSC accumulation in HCC tumors by blocking the AKT/NF-κB pathway, thereby inhibiting CXCL1/CXCL3-mediated chemotaxis [114]. These multi-layered interventions underscore the capacity of TCM to remodel the immunosuppressive TME in HCC, providing a robust foundation for enhancing anti-tumor immunity and overcoming therapeutic resistance.

### Targeting tumour-associated fibroblasts (CAFs)

CAFs represent the predominant mesenchymal cell population within the HCC tumor microenvironment. These cells play a pivotal role in promoting tumor growth, metastasis, and treatment resistance through the secretion of various growth factors and cytokines, which contribute to the establishment of an immunosuppressive milieu. By specifically targeting CAFs, TCM has demonstrated the potential to inhibit their tumor-promoting functions and reverse the immunosuppressive state within the TME.

#### Inhibition of CAFs activation

The activation of CAFs is predominantly regulated by the TGF-β/SMAD signaling pathway, which plays a central role in their differentiation and pro-tumorigenic functions. TGF-β, a key activator of CAFs, not only directly promotes their differentiation but also enhances their tumor-promoting capabilities through SMAD protein-mediated signaling. TCM has been shown to effectively inhibit CAFs activation by targeting the TGF-β signaling pathway. Norcantharidin (NCTD), a structurally modified derivative of norcantharidin [115, 116], significantly suppresses the activation of the TGF-β/Smad3 signaling pathway. This is achieved through the upregulation of FAM46C expression, which leads to a reduction in the relative phosphorylation levels of Smad2/3. Consequently, NCTD blocks the metastasis-promoting properties of CAFs in HCC [117]. Furthermore, recent studies have demonstrated that farrerol reduces the secretion of TGF-β1 by CAFs, thereby effectively inhibiting CAFs-induced HCC cell migration and EMT [118].

#### Inhibition of CAFs-mediated immunosuppression

By inhibiting the activation of CAFs, TCM can fundamentally reduce their tumour-promoting effects; furthermore, targeting the immunosuppressive factors secreted by CAFs can block their negative regulation of immune cells and achieve dual intervention.It has been found that factors such as TGF-β and CXCL11 secreted by CAFs not only promote tumour proliferation and metastasis, but also recruit immunosuppressive cells (e.g., M2-type macrophages) to form an immunosuppressive microenvironment [29, 30]. Herbal polysaccharide components reverse CAFs-mediated immunosuppression by enhancing CD8^+^ T cell activity [119]. In addition, CCL5 secreted by CAFs promotes metastasis by activating the HIF1α/ZEB1 axis in HCC cells, whereas herbal components such as glycyrrhizic acid may inhibit the interaction of CAFs with tumour cells and reduce the release of immunosuppressive factors [28, 120].

### Regulation of ECM remodelling

ECM is an important non-cellular component of the HCC tumour microenvironment and its remodelling plays a key role in tumour invasion and metastasis. TCM has demonstrated the ability to inhibit HCC metastasis by modulating ECM remodeling processes. Through targeted interventions, TCM can disrupt the structural and functional alterations of the ECM that promote tumor progression, thereby offering a promising therapeutic approach to mitigate HCC metastasis.

#### Inhibition of ECM deposition

Pathological overdeposition of collagen and fibronectin, the core components of ECM, has been shown to promote the metastatic process of HCC. This pathological mechanism suggests that TCM may slow down the progression of HCC by modulating the biosynthetic pathways of ECM components. Studies have shown that plant-derived active ingredients (e.g., flavonoids, alkaloids, etc.) can target the tumour stromal microenvironment and specifically inhibit the abnormal deposition of ECM. Herbal extracts represented by HA&GA-LPs nanoformulations can effectively reduce ECM deposition and tumour angiogenesis by inhibiting collagen biosynthesis, which in turn improves the physical barrier properties of the tumour microenvironment and ultimately inhibits the invasive metastatic potential of HCC cells [121]. Notably, the EMT process plays a key driving role in ECM remodelling. Traditional Chinese medicines, such as FZXZP, have been found to inhibit the EMT process, which is closely related to the overproduction of fibronectin, suggesting that it may indirectly inhibit fibronectin synthesis by regulating genes related to ECM remodelling [122]. Molecular mechanism studies further revealed that Centipeda minima (CM), an active ingredient of TCM, significantly inhibited the invasive migration ability of HCC cells by bi-directionally regulating the expression of cell adhesion molecules (down-regulation of the mesenchymal marker, N-cadherin/up-regulation of the epithelial marker, E-cadherin), thus blocking the pathological remodelling process of ECM, and ultimately reducing the abnormal ECM deposition [123]. In terms of ECM metabolic balance, MMPs family plays a central role in ECM degradation, when the protein hydrolysis activity of MMP (especially MMP-2/9) is inhibited, it will lead to the reduction of ECM decomposition and accumulation of components. Research on the mechanism of action of Fuzheng Xiezheng Formula (FZXZP) shows that the compound can block the pro-cancer effect of collagen degradation products by down-regulating the enzymatic activity of MMP-2/9 [122].

#### Enhanced degradation of ECM

As a key enzyme family for ECM degradation, the regulation of the expression level of MMPs directly affects the metabolic balance of ECM. Studies have shown that TCM may enhance ECM degradation by promoting the expression of specific MMPs isoforms [124]. For example, the Astragalus-atractylodis Macrocephalae drug pair (HQBZ) may inhibit pro-carcinogenic ECM remodelling by affecting the MMP/TIMP ratio, while promoting the clearance of fibrotic ECM [125, 126]. It is noteworthy that the same TCM component may exert bi-directional regulation of MMPs. For example, Panax ginseng may upregulate MMP-1 to degrade fibrotic ECM while inhibiting MMP-9 [127, 128]. In conclusion, TCM may inhibit HCC by up-regulating specific MMPs (e.g., MMP-1) to degrade excessive ECM at the fibrotic stage and reduce the fibrotic microenvironment during the inhibition of HCC. Meanwhile, in the tumour microenvironment, invasion may be inhibited by down-regulating pro-metastatic MMPs (e.g. MMP-9). Therefore, there is a need to clarify the bi-directional regulation of MMPs by TCM at different pathological stages and the specific mechanism of action.

Tissue inhibitors of matrix metalloproteinases (TIMPs), a family of endogenous inhibitors of MMPs, maintain dynamic homeostasis of ECM metabolism by antagonising MMPs activity. The key active ingredients of Wuzhu Tang could affect the ECM remodelling process by targeting the regulation of the MMP-TIMP axis, and the mechanism may involve selective inhibition of the expression levels of TIMP1/2 [129, 130]. In addition, the TGF-β/SMAD pathway is an important target for the regulation of TIMPs by Chinese medicines. Certain herbal extracts reduce the expression of TIMP1 and TIMP2 by inhibiting this pathway, thereby deregulating the inhibition of MMPs and promoting ECM degradation [115].

### Inhibition of angiogenesis

#### Inhibition of angiogenic factor expression

Overexpression of angiogenic factors is a central trigger for abnormal vascular proliferation in HCC. Some Chinese herbal components have demonstrated anti-angiogenic effects. Eupafolin, a flavonoid extracted from Salvia divinorum, significantly inhibited the secretion of VEGF in HepG2 in a dose-dependent manner, and showed potent anti-angiogenic and anti-tumour activity in HCC [131]. Fei Yao et al. demonstrated for the first time that serpentin could down-regulate the expression of VEGF and NF-κB p65 and upregulate the expression of IκB-α in tumours and paracancerous tissues, thereby inhibiting angiogenesis in an in situ mouse model of HCC [132]. Purified vitexin compound 1 (VB-1) inhibited the proliferation of HCC cells and also reduced the secretion of VEGF, inhibited the formation of endothelial tubules, and inhibited the growth and angiogenesis of HCC by inactivating Akt and activating FOXO3a [133]. Celastrus orbiculatus extract (COE), a mixture of 11 terpenoids isolated from the herb Celastrus orbiculatus vine, can block TGF-β 1-induced VM formation and HCC tumour growth by down-regulating Notch1 signaling and is considered superior to other anti-angiogenic drugs due to its It is considered a promising candidate for HCC treatment because of its superiority over other anti-angiogenic drugs [134]. Hydroxy saffron yellow A (HSYA), an active ingredient of the herb Carthamus tinctorius L. (safflower), significantly reduced the levels of MMP-2 and MMP-9 in tumour tissues of mice transplanted with H22 and inhibited the phosphorylation of p38MAPK in a concentration-dependent manner, thereby inhibiting angiogenesis in HCC [135].

#### Inhibition of vascular endothelial cell proliferation and migration

Angiogenesis not only relies on the secretion of pro-angiogenic factors, but also requires the proliferation and migration of endothelial cells to complete the construction of new blood vessels. TCM can directly block the angiogenic process by targeting the key aspects of endothelial cell behaviour. A Chinese medicine compound, Sanshi Cat (SSM), significantly inhibited the angiogenic ability of HCC-induced endothelial cells (EA.hy926 cells) in a simulated tumour hypoxic environment, suggesting that it may interfere with endothelial cell activity by regulating hypoxia-related signaling pathways [136]. In addition, some herbal medicines indirectly inhibit endothelial cell activation by reducing pro-angiogenic factors (e.g. VEGF) secreted by tumour cells. For example, flavonoid components (e.g., baicalein, hanhuangqin) in Scutellaria baicalensis extracts have been shown to inhibit VEGF expression, thereby reducing the proliferation and migration of endothelial cells [137].

## Herbal medicines with potential to modulate the microenvironment of HCC

### Individual Chinese medicines and their active ingredients

#### Berberine

Berberine (BBR), as a core active ingredient in TCM such as Rhizoma Coptidis, exhibits a multidimensional role in HCC microenvironment regulation. Experimental studies demonstrated that BBR significantly inhibited tumour growth in an in situ mouse model of HCC in a dose-dependent manner, and the mechanism involved a systematic remodelling of the immune microenvironment: on the one hand, BBR enhanced the anti-tumour immune response by inducing macrophage polarisation towards the M1-type, and on the other hand, when coupled with NK92-MI cells, it up-regulated the expression of perforin and granzyme B through up-regulation of the NK cells'killing efficiency of SMMC-7721 and Hep3B cells to enhance the killing efficiency and induce apoptosis of tumour cells [138, 139]. In addition, BBR may block invasive metastasis of HCC by inhibiting the PI3K/AKT/mTOR signaling pathway and modulating ECM-related molecules, and significantly reversed drug resistance after combination with sorafenib [140, 141]. To address the bottleneck of low BBR bioavailability, nano-delivery systems (e.g., BBR-NPs) showed enhanced anticancer efficacy in AFB1-induced HCC models by improving hepatic targeting [142].

However, the clinical application of BBR still faces challenges: its multi-target mechanism of action needs to be resolved with the help of technologies such as single-cell sequencing (e.g., patients with high expression of SH3D21 may be more sensitive), and individualised therapeutic regimens based on epigenetic markers such as 5-methylcytosine (5mC) need to be developed [143, 144]. Future studies need to further explore the regulatory effects of BBR on CAFs and exosomes and optimise the pharmacokinetic properties through structural modifications in order to facilitate the leap from the laboratory to the clinic for this natural drug [91, 145, 146].

#### Cinobufagin

As a traditional Chinese medicinal preparation derived from the dried skin glands of the toad Bufo toad, Huachansu, with its 21 core active ingredients (e.g., the toad venom ligand analogues Bufalin and Cinobufagin), intervenes in the microenvironment of HCC through a multi-targeting mechanism [147]. Experimental studies demonstrated that huachansu inhibited HCC cell proliferation in a dose- and time-dependent manner and significantly reduced tumour volume and promoted HCC cell apoptosis in a DEN-induced HCC rat model [148]. In addition, Cinobufacini can prevent HepG2 cell migration and invasion by inhibiting EMT through the c-Met/ERK signaling pathway [149].

In terms of clinical application, huachansu has been widely used in the treatment of intermediate and advanced HCC since the 1970 s, and its injection can reduce the size of the tumour, which is in line with the theory of Chinese medicine of ‘invigorating blood circulation and removing blood stasis’ [150]. In combination therapy, huachansu synergistically with transarterial chemoembolisation (TACE) reversed hypoxic microenvironment-induced treatment resistance and effectively prolonged PFS and OS in patients with unresectable HCC [151]. However, the in-depth transformation of huachunin faces a triple challenge: Firstly, the mechanism of action of huachunin has not been fully resolved due to its multi-component nature, and the specific regulation of TME cell subpopulations needs to be revealed with the help of single-cell sequencing technology. Secondly, the standardisation of the formulation needs to be resolved, and the difference in Bufalin content between different batches of injections may affect the consistency of efficacy. Thirdly, the existing clinical evidence is mostly from observational studies, and multi-centre phase III trials are needed to verify its synergistic potential with emerging therapies (e.g. CAR-T, bispecific antibodies) [152]. Future research needs to focus on the development of nano-delivery systems to improve targeting and reveal the spatial and temporal features of their regulation of TME heterogeneity through spatial transcriptomics to provide scientific support for the modernisation and transformation of this traditional medicine.

#### Huangqin

Huangqin (Scutellaria baicalensis Georgi) and its flavonoid active ingredients (e.g. Baicalin, Baicalein) exhibit systemic anti-tumour potential from molecular mechanisms to clinical translation through multi-dimensional regulation of the HCC microenvironment. Experimental studies showed that baicalin significantly reversed the immunosuppressive microenvironment by inducing immunogenic cell death (ICD) in HCC cells and activating adaptive immune response; meanwhile, it alleviated the inhibitory effect of the acidic microenvironment on lymphocytes by inhibiting the activity of lactate dehydrogenase A (LDHA) and lowering the concentration of lactate in TME [153, 154]. Network pharmacological analyses further revealed that baicalein inhibited HCC cell invasion by down-regulating the AKR1B10/PI3K-Akt pathway [155].

In clinical applications, Scutellaria baicalensis compound (e.g., HQZX soup) combined with TACE in the treatment of patients with intermediate- to advanced-stage HCC resulted in prolonged median progression-free survival, and its above-ground parts were found to be rich in active components such as wild baicalin by metabolomics, suggesting the potential for the development of stem and leaf resources [156, 157]. However, the multi-targeted nature of Baicalin has led to the fact that its direct molecules of action (e.g., immune checkpoint regulatory targets) have not been fully resolved, and the available clinical evidence is mostly based on small-sample observational studies [158, 159]. In the future, it is necessary to reveal the spatial and temporal regulation of TME heterogeneity by Scutellaria baicalensis components with the help of spatial transcriptomics, and develop baicalin nanocrystal preparations to break through the translational bottleneck, as well as to validate its synergistic effect with PD-1 inhibitors through multicentre phase III clinical trials, so as to promote this TCM to make the leap from laboratory evidence to the practice of precision medicine.

### Compound prescription of Chinese medicine

#### Xiao Chai Hu Tang

Xiao Chai Hu Tang (XCHT), as a classic TCM compound, demonstrates multidimensional therapeutic potential through its multi-component properties (Chai Hu, Scutellaria baicalensis, Semen Heterophyllum, etc.) by synergistically modulating the immune, inflammatory, and fibrotic processes in the HCC microenvironment. Experimental studies have shown that the self-emulsifying nanophase of XCHT can be targeted and delivered to the liver, significantly reducing ALT levels in a CCl_4_-induced hepatic fibrosis-HCC mouse model, as well as alleviating chronic inflammation and decreasing collagen deposition by inhibiting TNF-α and IL-6 secretion [160, 161]. In terms of anti-tumour progression, the active ingredients of XCHT (e.g. Chaihu saponin, baicalein) block HCC cell invasion and metastasis by inhibiting MMP-9 expression and VEGF-mediated angiogenesis, and its combination with sorafenib synergistically reduces tumour cell viability [124, 162].

Clinical observations showed that HQZX compound containing XCHT combined with TACE in the treatment of patients with intermediate and advanced HCC resulted in prolonged median survival and may improve patients'quality of life by modulating IL-17 and TGF-β pathways [156, 163]. However, its multi-targeted mechanism of action still requires the use of spatial transcriptomics and other technologies to resolve the component-microenvironment interaction network, while the existing clinical evidence is mostly based on retrospective studies, and multi-centre randomized controlled trials are urgently needed to validate standardized protocols. Future studies could explore its synergistic effect with immune checkpoint inhibitors and screen sensitive populations through metabolomics, so as to promote the precise application of this millennia-old formula in modern tumour microenvironment therapy.

#### Fuzheng Jiedu decoction

Fuzheng Jiedu Decoction demonstrates comprehensive anti-tumour potential by regulating the HCC microenvironment at multiple levels [164]. Experimental studies have shown that Fuzheng Jiedu Decoction significantly impairs the self-renewal ability of HCC tumour stem cells by inhibiting the Wnt/β-catenin pathway under hypoxic conditions, thereby reducing the risk of tumour recurrence and metastasis [165]. In combination therapy with TACE, Fuzheng Jiedu Decoction not only enhanced the killing efficiency of chemotherapeutic agents on HCC cells, but also alleviated chemotherapy-induced hepatotoxicity and myelosuppression by modulating the PI3K/AKT pathway [103, 166].

In clinical application, postoperative adjuvant use of Fuzheng Jiedu Decoction significantly reduced the recurrence rate of HCC patients and significantly inhibited metastasis formation by down-regulating MMP-9 and TIMP-1 when combined with PD-1 inhibitor therapy [167, 168]. These studies provide a theoretical basis for the precise application of Fuzheng Jiedu Decoction in the treatment of HCC, which is particularly suitable as an adjunct to combination therapy.

#### Biejia Jianwan

Biejia Jianwan and its modified formulas (e.g., M-BJJW) demonstrate systemic effects in immunomodulation, antifibrotic and antitumour synergistic therapies through multidimensional modulation of the HCC microenvironment. Experimental studies demonstrated that M-BJJW significantly elevated the proportion of cytotoxic T lymphocytes (CTLs) in a DEN-induced rat HCC model, while simultaneously decreasing the abundance of Tregs and the levels of immunosuppressive factors, such as IL-6 and IL-10, and that the core mechanism involves down-regulating the expression of PD-L1 by inhibiting the HIF-1α/STAT3/NF-κB signaling pathway, thereby blocking the immune escape of tumour cells [169]. Entecavir plus Biejia-Ruangan compound reduces the risk of hepatocellular carcinoma in Chinese patients with chronic hepatitis B [170, 171].

Clinical applications have shown that the detoxification, elimination of stasis and nourishment of yin formula containing turtle shell ingredient as postoperative adjuvant therapy can reduce postoperative recurrence and metastasis and prolong the survival of patients with HCC [165]. In addition, M-BJJW may reverse immune escape in HCC by down-regulating PD-L1 expression, and has potential for use in combination with PD-1/PD-L1 inhibitors [169]. In conclusion, Biejia Jianwan regulates the HCC microenvironment through multi-targets and multi-pathways, and its clinical translation requires further standardisation of the formulation and the conduct of large-sample randomised controlled trials.

## Integration of TCM with conventional therapies for HCC treatment

### TCM combined with immunotherapy

Immunotherapy, particularly immune checkpoint inhibitors (e.g., PD-1/PD-L1 antibodies), has made significant progress in the treatment of HCC. However, the overall response rate to immunotherapy is low and some patients experience immune-related adverse effects. TCM can have a synergistic effect with immunotherapy by modulating TME and enhancing the body's immune function.

#### Enhancing the efficacy of immune checkpoint inhibitors

TCM reverses the immunosuppressive state by modulating immunosuppressive cells (e.g. regulatory T cells, myeloid-derived suppressor cells) and promoting the infiltration of immune effector cells (e.g. cytotoxic T cells) in the immune microenvironment of HCC [172, 173]. For example, Shenlian Decoction (SLD) enhances the anti-tumour activity of T cells by inhibiting the expression of immune checkpoint molecules (e.g. PD-L1) [174]. In addition, the polysaccharide component of TCM activates dendritic cells and macrophages, promotes antigen presentation, and improves the immune response of ICIs [119]. Notably, HCC resistance to ICIs is associated with T-cell depletion and low immunogenicity in the immune microenvironment [175, 176]. TCM such as the Astragalus-Quercus alba drug pair (HQBZ) restores T-cell function and improves the objective remission rate of ICIs by down-regulating the PD-1/PD-L1 axis [125, 177].

#### Mitigating immunotherapy-related adverse effects

Immunotherapy may lead to immune over-activation, triggering immune-related adverse reactions (e.g., colitis). TCM can mitigate immunotherapy-related adverse reactions by regulating immune homeostasis. For example, for ICIs-induced immune-related adverse reactions such as colitis, TCM can reduce the inflammatory response and improve the safety of treatment [177]. In addition, HCC patients are often associated with hepatic insufficiency, and immunotherapy may further exacerbate hepatic impairment. TCM can mitigate the adverse effects of immunotherapy by protecting liver function. For example, Huachansu Tablet (HCS) has been shown to alleviate treatment-related liver injury in clinical studies [148]. In conclusion, TCM not only enhances the efficacy of ICIs but also improves treatment tolerance through multidimensional intervention in the immune microenvironment of HCC, providing a new strategy for the comprehensive treatment of advanced HCC.

### TCM combined with targeted therapy

Targeted therapies (e.g. sorafenib and lenvatinib) are first-line treatments for advanced HCC, but they have limited efficacy and are susceptible to drug resistance. TCM can be synergistic with targeted therapies by modulating the TME and increasing the sensitivity to targeted drugs.

#### Improving the sensitivity of targeted drugs

The active ingredients in TCM (e.g., Artemisia annua and bitter ginseng extracts) can inhibit EMT and cancer stem cell activity by modulating pathways such as NF-κB and HIF-1, thereby reversing the resistance of HCC to targeted drugs (e.g., sorafenib) [178–180]. For example, network pharmacological analyses have found that TCM core components enhance tumour immunogenicity through the PD-L1 immune-associated pathway and increase the sensitivity of targeted drugs [181]. TCM complexes (e.g. SPXJF, Huanchansu) have more comprehensive regulatory effects than single-target drugs through multiple active ingredients (e.g. polysaccharides, alkaloids) acting simultaneously on different targets (e.g. apoptosis, autophagy, cell cycle blockade) in the HCC microenvironment [182–184]. For example, TCM active ingredients interfere with tumour signaling and enhance the sensitivity of targeted drugs by modulating the ceRNA network (e.g. lncRNA/circRNA) [179]. Combination of ginsenoside Rg3 (Rg3), Solamargine (SM), Bushen Jianpi (BSJP) and Sorafenib (SFN) inhibits the growth of HCC and enhances the anticancer effect of Sorafenib [185–187].

#### Mitigating adverse effects of targeted therapies

Targeted drugs (e.g. sorafenib) inhibit tumour growth but are often accompanied by cardiovascular side effects such as diarrhoea, hypertension and myocardial damage [188]. These adverse effects limit therapeutic tolerability, and TCM alleviates such problems through holistic modulation. Meta-analysis showed that TCM combined with targeted therapy significantly reduced the incidence of diarrhoea and hypertension and improved patients'quality of life [189]. In addition, retrospective studies have found lower rates of cardiovascular events in HCC patients on long-term TCM use, possibly related to improved mitochondrial function [190]. For example, flavonoids improve myocardial microcirculation, reduce oxidative stress and protect the cardiovascular system [191]. In conclusion, TCM provides a safe and cost-effective adjuvant strategy for clinical use by enhancing anti-tumour effect and effectively reducing cardiovascular toxicity through multi-target synergism in HCC-targeted therapy.

### TCM combined with chemotherapy/radiotherapy

Chemotherapy and radiotherapy are important treatments for HCC, but their efficacy is limited and their toxic side effects are large. TCM can have a synergistic effect with chemotherapy/radiotherapy by enhancing their efficacy and reducing their toxic side effects.

#### Enhancing the efficacy of chemotherapy/radiotherapy

TCM can enhance the efficacy of chemotherapy/radiotherapy by modulating TME and enhancing the sensitivity of tumour cells to chemotherapy/radiotherapy. Aidi injection (AD), epimedium, dihydroartemisinin, and Dahuang zhechong pill attenuated the cardiotoxicity of DOX, further inhibited tumour formation in loaded mice by promoting apoptosis and inhibiting the proliferation of HCC cells, and increased the sensitivity of DOX for the treatment of HCC [192–195]. TCM components (e.g., locust ear extract) increase radiotherapy-induced DNA damage accumulation by inhibiting the EGFR-mediated DNA repair signaling pathway, thereby increasing radiotherapy sensitivity [196].

#### Reducing the toxic effects of chemotherapy/radiotherapy

Chemotherapy/radiotherapy may lead to bone marrow suppression, triggering leukopenia and anaemia. TCM can alleviate the toxic side effects of chemotherapy/radiotherapy by protecting bone marrow function. Neutral polysaccharide of Panax ginseng (NPPN), Shen-Ling-Bai-Zhu Powder (SLBZP), and He-Wei Granule can enhance CTX treatment, promote apoptosis of HCC cells, effectively reduce the toxicity of CTX, and prolong the survival of the loaded mice [197–199].

**Fig. 3:**
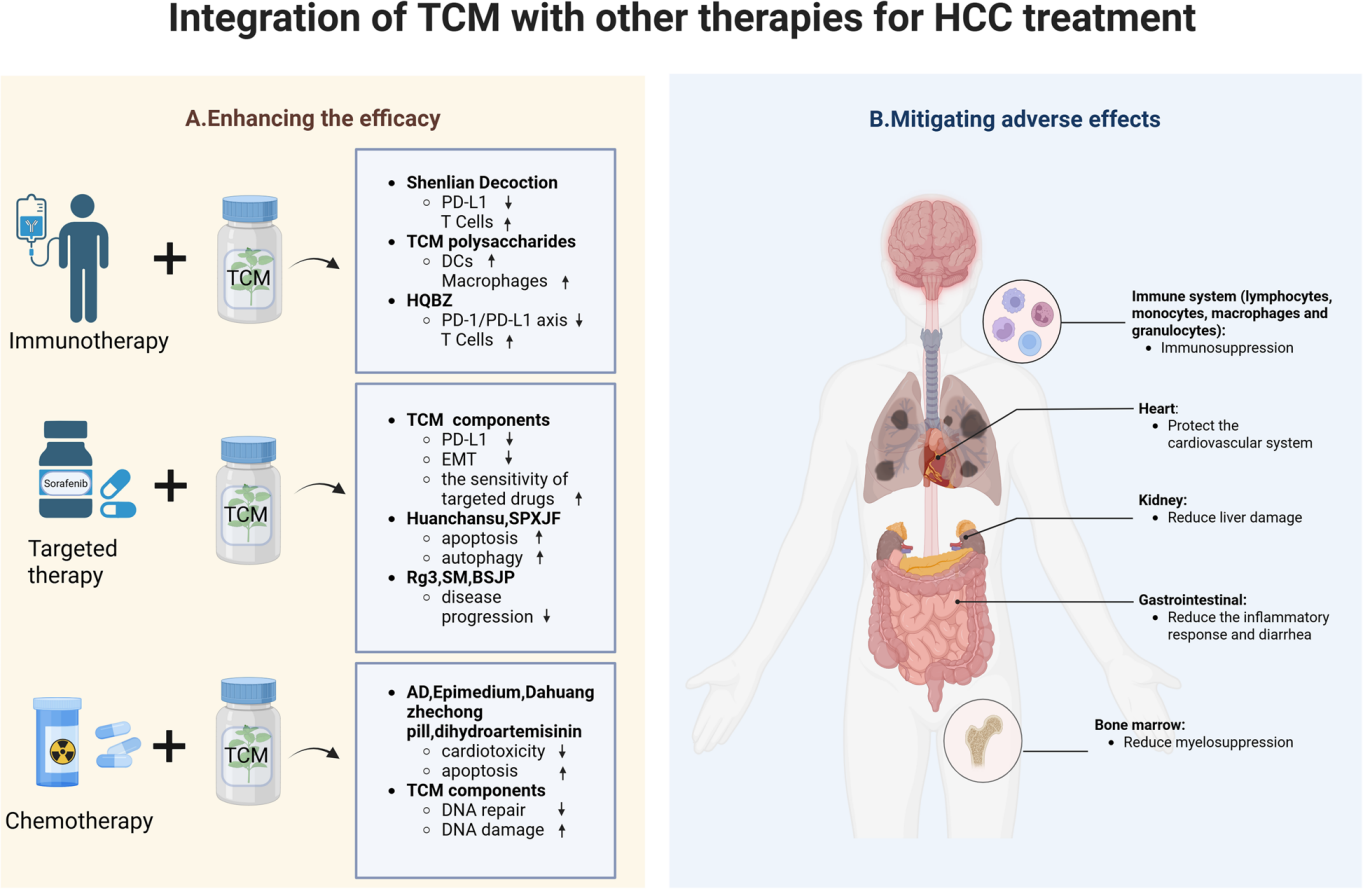
Proposed model for integration of TCM into HCC therapeutic strategies. **A** TCM in combination with immunotherapy, targeted therapy and chemotherapy can enhance the efficacy of the drug. **B** TCM in combination with immunotherapy, targeted therapy and chemotherapy can mitigate the adverse effects of western drugs

TCM emphasizes holistic concepts and individualized treatment, aligning with the precision medicine approach of modern medicine. In the treatment of HCC, the unique advantages of TCM provide opportunities for integrating traditional and Western medicine. This paper proposes a model for incorporating TCM into HCC treatment (as shown in Fig. 3), aiming to achieve more comprehensive therapeutic outcomes through the integration of traditional and Western medicine.

## Challenges and future perspectives

### Limitations and obstacles in studying the microenvironment in HCC

The HCC microenvironment is complex, and this complexity makes it difficult to study the HCC microenvironment and to isolate and analyse the roles of the various components. First, the HCC microenvironment consists of multiple cell types (e.g., hepatic stellate cells, immune cells, liver endothelial cells, etc.), and the crosstalk between the different cell types promotes HCC proliferation and progression, immune escape, and angiogenesis. Currently, most studies have focused on the mechanistic exploration of signaling pathways and molecular mechanisms associated with malignant progression of HCC in a single environment, with a single stimulus and a single research target, whereas the complex cellular crosstalk in the HCC microenvironment results in the continuous remodelling of the TME, which in turn generates resistance to treatment [200]. Secondly, HCC tumours have significant heterogeneity between patients and within tumours of the same patient, which is a key determinant of the differences in prognosis and drug sensitivity of patients with advanced HCC, ultimately leading to the difficulty of establishing a unified experimental model and therapeutic strategy in tumour therapy [201]. In addition, currently available animal models often fail to fully mimic the biology of human HCC, somewhat limiting in-depth studies of the role of the microenvironment [202]. Also, it is worth noting that there are multiple immune escape mechanisms in the HCC microenvironment that allow the tumour to resist attack by the immune system. It is currently believed that immunosuppressive TME contributes to immune escape in HCC, which is mainly classified into 1) immunosuppressive cells; 2) co-inhibitory signals; 3) soluble cytokines and signaling cascades; 4) metabolically hostile tumour microenvironment; and 5) intestinal microbiota affecting the immune microenvironment [203]. This further adds to the complexity of understanding immune interactions. In addition, metabolic remodelling of HCC cells and the tumour microenvironment affects tumour growth and metastasis, adding to the difficulty of the study [72]. Notably, current clinical evidence supporting TCM's modulation of the HCC microenvironment faces methodological challenges. Most existing studies are limited by small sample sizes, single-center designs, and insufficient long-term follow-up [204].The lack of standardized outcome reporting (e.g., inconsistent use of RECIST criteria vs. TCM syndrome evaluation) and potential confounding factors (e.g., concurrent use of Western therapies) further compromise the reliability of conclusions [205, 206]. These have led to limitations in gaining an in-depth understanding of the HCC microenvironment and its impact on tumourigenesis, progression and treatment response.

### Future directions and research opportunities

**Fig. 4:**
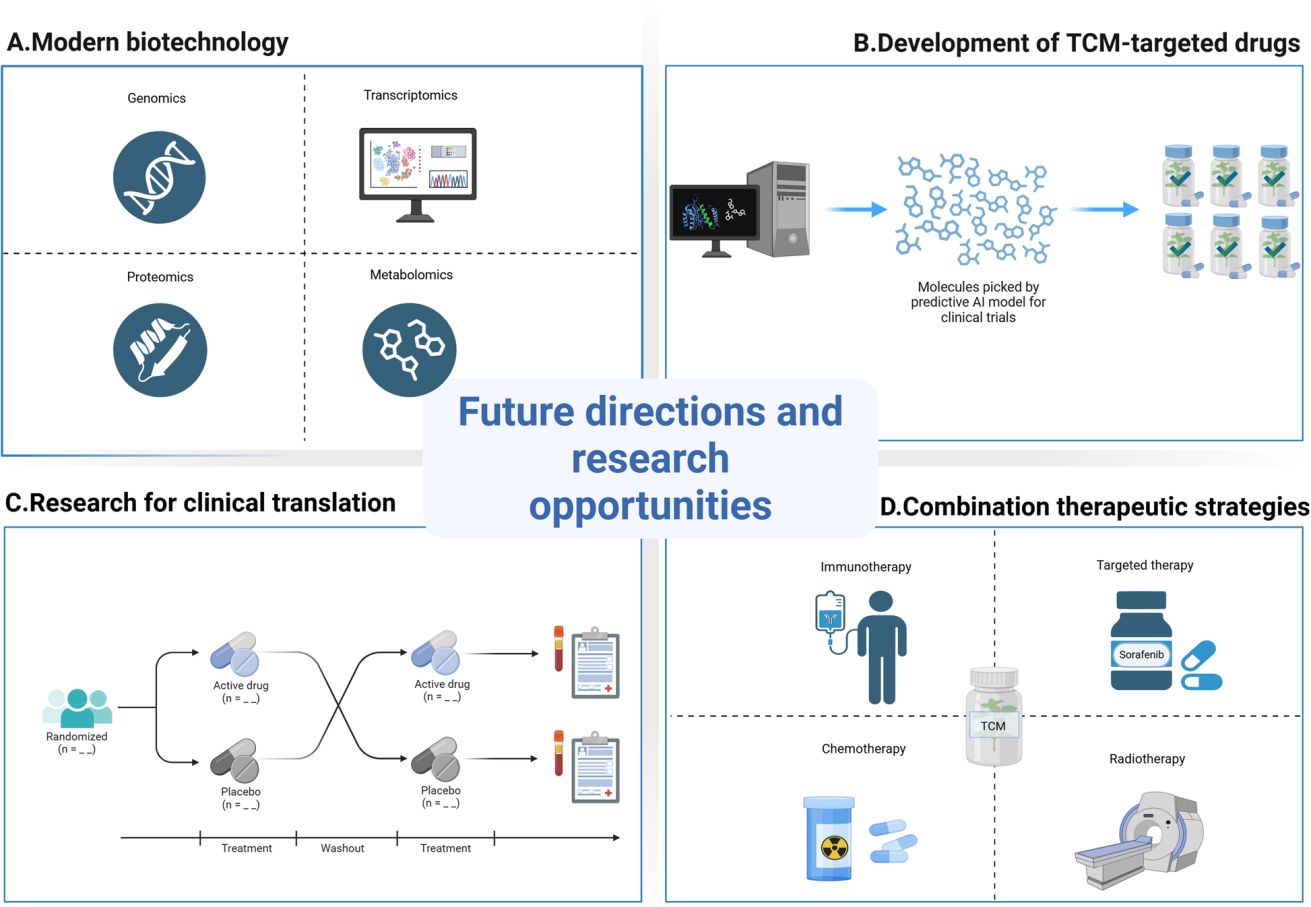
Future directions and research opportunities. **A** Modern biotechnology (e.g. genomics, transcriptomics, proteomics and metabolomics) was used to study the potential mechanisms by which TCM constituents regulate TME. **B** Developing Chinese medicinal preparations for specific targets in the HCC microenvironment to improve their selectivity and effectiveness in regulating TME and to achieve precision therapy. **C** To carry out high-quality RCTs, to establish a multidimensional evaluation system covering immune function and quality of life, and to promote the application and development of TCM in the treatment of HCC. **D** Combine TCM with modern medicine, evaluate the effects of their joint application, and explore the potential of TCM in improving treatment effects and reducing side effects

The occurrence and development of HCC are regulated by the complex microenvironment, and the synergistic multi-target and multi-pathway role of TCM in regulating TME is becoming a research hotspot. However, the following problems still exist: 1) The mechanism of action needs to be studied in depth. In-depth exploration of the mechanism of action of TCM in regulating the HCC microenvironment is the key, using modern biotechnology (e.g. genomics, transcriptomics, proteomics and metabolomics) to study how TCM components affect the function of immune cells, cytokines and tumour-associated fibroblasts within the TME, so as to reveal their potential anti-tumour mechanisms. 2) Development of TCM-targeted drugs. The greatest advantage of the efficacy of TCM lies in the synergistic effect of multiple components and targets, and the development of TCM preparations for specific targets in the HCC microenvironment will become an important direction of research by optimising the TCM components and enhancing their selectivity and effectiveness in regulating the TME to achieve precise treatment. 3) Application of modern drug delivery systems. Targeted nano-preparation has the function of improving the solubility, stability, permeability and half-life characteristics of active drugs, effectively improving the bioavailability and bioactivity of the active ingredients of Chinese herbal medicines, and targeting the tumour tissues or tumour cells [207]. The use of advanced drug delivery systems (e.g., nanomedicines) can improve the homoeopathic and putative properties of herbal components in liver cancer therapy. 4) Combination therapy strategies. Combine TCM with modern medicine, evaluate the effects of their joint application, and explore the potential of TCM in improving treatment effects and reducing side effects. This integrated treatment model is expected to improve the prognosis and quality of life of patients [15, 180]. 5) Rigorous clinical translation research. High-quality randomised controlled trials (RCTs) should be conducted with multicenter designs (target ≥ 200 participants for phase III trials) and extended follow-up (≥ 36 months) to capture long-term outcomes. Studies must standardize outcome assessments by integrating Western criteria (e.g., mRECIST, overall survival) with TCM-specific evaluations (e.g., syndrome differentiation scores), implement stratified randomization based on TME biomarkers (e.g., cytokine profiles), and apply blinding procedures for subjective endpoints. Evidence synthesis frameworks should be established through consortiums to develop core outcome sets and promote individual participant data meta-analyses, particularly for rare HCC subtypes. In conclusion, the study of TCM in the microenvironment of HCC has a broad prospect. To bridge the gap between preclinical promise and clinical validation, the application and development of TCM in HCC treatment should be advanced through mechanism exploration guided by multi-omics technologies, combinatorial treatment optimization, targeted drug delivery systems, and rigorously designed clinical trials adhering to CONSORT-TCM extension guidelines (as shown in Fig. 4).

## Conclusion

### Recap of the importance of understanding the microenvironment in HCC

TME refers to the internal environment surrounding the tumour cells and affecting their survival and development, which is composed of a variety of cell types (e.g., tumour cells, immune cells, fibroblasts, etc.) and biomolecules (e.g., growth factors, cytokines, etc.). This environment is closely related to the process of tumour genesis, growth, invasion and metastasis, and involves not only the structure, function and metabolic state of the tissues in which the tumour is located, but also the intrinsic environment of the tumour cells (e.g., cell nucleus and cytoplasm). While conventional tumour therapy focuses on the tumour cells themselves, with the deeper exploration of tumour mechanisms, the therapeutic focus has gradually shifted to the interactions between the tumour and the surrounding tissues. The complex cellular crosstalk induced by cellular and non-cellular components of the TME contributes to the formation of an immunosuppressive TME, which facilitates immune escape for the tumour cells, while at the same time promotes angiogenesis to satisfy the tumour's nutritional needs. An in-depth understanding of these features of the microenvironment can help develop new therapeutic strategies, such as reversing immunosuppression, restoring the anti-tumour activity of the immune system, or inhibiting tumour angiogenesis, which can effectively inhibit tumour growth and metastasis, and improve patient survival. Therefore, an in-depth study of the mechanism of action of the microenvironment of HCC is of great significance for the discovery of more effective therapeutic approaches and the improvement of patient prognosis.

### Potential of TCM in modulating the HCC microenvironment and improving patient outcomes

TCM has demonstrated remarkable potential in regulating the microenvironment of liver cancer (especially HCC), and has become an important adjuvant for improving the prognosis of liver cancer patients. Its unique regulatory mechanism can deeply affect multiple cell types and biomolecules in the microenvironment, thereby altering the tumour growth environment. By regulating the function of immune cells in the microenvironment, TCM is able to regulate the function of immune cells in the microenvironment, alleviate the state of immunosuppression and enhance the anti-tumour activity of the immune system. By promoting the proliferation and differentiation of immune cells, TCM is able to increase the recognition and attack on tumour cells, thus inhibiting the growth and metastasis of tumours. At the same time, TCM can also inhibit tumour angiogenesis and reduce the nutrient supply to tumours, further curbing tumour development. In addition, TCM can promote apoptosis and necrosis of tumour cells by regulating cell signaling, metabolic state and other mechanisms in the microenvironment, thus accelerating the death of tumour cells. This multi-target and multi-pathway therapeutic approach makes TCM have significant advantages and potentials in treating the microenvironment of liver cancer. More importantly, the multi-component and multi-target characteristics of TCM enable it to precisely intervene in the complex mechanisms in the microenvironment, such as promoting apoptosis of tumour cells and regulating the balance of cytokines, thus improving the microenvironment in a multi-dimensional way and opening up a new pathway for liver cancer treatment. This kind of holistic and comprehensive treatment not only enhances the therapeutic effect of liver cancer, but also significantly improves the quality of patients'survival, bringing longer survival and better quality of life to liver cancer patients. In conclusion, the potential of TCM in treating the microenvironment of HCC should not be ignored, and its unique regulatory mechanism and comprehensive therapeutic effect provide new therapeutic options and hope for HCC patients, and it is expected to make an important contribution to the improvement of patients'prognosis and quality of life.

## Data Availability

No datasets were generated or analysed during the current study.

## Abbreviations

HCC: Hepatocellular Carcinoma
TME: Tumor microenvironment
TCM: Traditional Chinese Medicine
WHO: World Health Organization
HBV: Hepatitis B virus
HCV: Hepatitis C virus
NAFLD: Nonalcoholic fatty liver disease
TACE: Transarterial chemoembolization
ECM: Extracellular matrix
CAFs: Cancer-Associated Fibroblasts
DCs: Dendritic Cells
NKs: Natural Killer Cells
MDSCs: Myeloid-Derived Suppressor Cells
TAMs: Tumour-associated macrophages
HSC: Hepatic stellate cells
HIF: Hypoxia-inducible factor
Tregs: Regulatory T cells
EMT: Epithelial-mesenchymal transition
EndMT: Endothelial-mesenchymal transition
MSCs: Mesenchymal stem cells
VEGF: Vascular endothelial growth factor
HIF-1α: Hypoxia-inducible factor-1α
PDGF: Platelet-derived growth factor
FGF: Fibroblast growth factor
a-HSC: Activated haematopoietic stem cells
TECs: Tumour vascular endothelial cells
TIMPs: Tissue inhibitors of matrix metalloproteinases
MMPs: Matrix Metalloproteinases
LPS: Lipopolysaccharide
TILs: Tumour infiltrating lymphocytes
arg-1: Arginase 1
PGE2: Prostaglandin E2
APCs: Antigen-presenting cells
VB-1: Vitexin compound 1
COE: Celastrus orbiculatus extract
